# LncRNA SNHG14 Regulated by ZNF460 Promotes Gastric Cancer Progression and Metastasis by Targeting the miR‐206/FNDC3A Axis

**DOI:** 10.1111/jcmm.70652

**Published:** 2025-06-16

**Authors:** Bin Liu, Tingting Lu, Guangming Zhang, Xiaohua Dong, Miao Yu, Hui Cai

**Affiliations:** ^1^ The First School of Clinical Medicine Lanzhou University Lanzhou China; ^2^ NHC Key Laboratory of Diagnosis and Therapy of Gastrointestinal Tumor Gansu Provincial Hospital Lanzhou China; ^3^ Key Laboratory of Molecular Diagnostics and Precision Medicine for Surgical Oncology in Gansu Province Gansu Provincial Hospital Lanzhou China; ^4^ Institute of Basic Research in Clinical Medicine China Academy of Chinese Medical Sciences Beijing China; ^5^ Phase I Clinical & Research Ward Gansu Provincial Hospital Lanzhou China

**Keywords:** ceRNA, gastric cancer, omics, SNHG14, transcription factor

## Abstract

The current study investigated the functional role of long non‐coding RNA SNHG14 (lncRNA SNHG14) in gastric cancer (GC) progression and its underlying mechanisms. Compared with para‐carcinoma tissues, SNHG14 was upregulated in GC tissues, correlating with a poor prognosis in GC patients. SNHG14 knockdown significantly weakened the proliferation, migration and invasion capabilities of GC cell lines while enhancing the apoptosis ability of GC cells. Simultaneously, SNHG14 overexpression reversed these effects. RNA fluorescence in situ hybridization (FISH) and nucleocytoplasmic separation assays revealed that SNHG14 was primarily located in the cytoplasm of GC cells. Combined sequencing of the miRNAome and transcriptome depicted that miR‐206 could be a potential target for SNHG14. Mechanistically, assays such as luciferase reporter, RNA immunoprecipitation (RIP) and RNA pulldown established that lncRNA SNHG14 acted as a sponge for miR‐206. This prevented the degradation of its target gene, FNDC3A, playing a tumour‐suppressive role in GC. In addition, FNDC3A directly interacted with the SNHG14 promoter and induced transcription, thus facilitating GC progression. Therefore, our research findings suggested a novel pathway to promote GC progression through the FNDC3A/lncRNA SNHG14/miR‐206/FNDC3A axis. Moreover, the findings indicated that SNHG14 could become a potential biomarker and therapeutic target for GC.

## 1 Introduction

Among the malignant tumours of the digestive system, gastric cancer (GC) possesses the highest incidence and fatality rates, second only to colorectal cancer. It poses a serious risk to the health and lives of people, ranking fifth among all cancer types based on incidence and mortality [1, 2]. Despite the advent of new therapies for GC, such as immunotherapy and targeted therapy, the prognosis of patients remains poor because of late diagnosis in most cases, leading to poorer enhancement of treatment outcomes [3]. Therefore, a deeper comprehension of the molecular mechanisms underlying GC could help identify novel therapeutic targets [4]. An increasing body of evidence indicates that long non‐coding RNAs (lncRNAs) function as tumour suppressors or oncogenes in cancer, influencing the proliferation, metastasis and apoptosis of cancer cells [5].

LncRNAs typically exert their biological functions by directly or indirectly regulating the expression of potential target genes at the epigenetic modification, transcriptional and post‐transcriptional levels [6, 7]. Small nucleolar RNA host gene 14 (SNHG14) is a widely expressed lncRNA, garnering the attention of several researchers. It may be involved in the intracellular regulatory process as a competing endogenous RNA (ceRNA) [8, 9]. The working principle behind competing endogenous RNAs depends on their ability to bind to microRNA response elements (MREs), thereby affecting the gene silencing induced by microRNAs [10]. In cancer research, several research findings have established that SNHG14 is closely associated with the malignant behaviours of cancer cells, mainly playing a non‐negligible role in enhancing their migration and invasion [11, 12]. MicroRNA‐206 (miR‐206) linked with SNHG14 is from the microRNA (miRNA) family [13]. The gene of miR‐206 is specifically located in the human chromosome 6 region, between the interleukin‐17 and polycystic kidney and hepatic disease 1 genes [14]. In studies on multiple cancer types, miR‐206 exhibits a significant tumour‐suppressive function. For instance, Deng's research indicated that miR‐206 could directly interact with the 3′ untranslated region (3′UTR) of the MUC1 gene, suppress its expression and exert effective anti‐tumour activity by inhibiting MUC1 expression [15]. Moreover, the protein encoded by the FNDC3A (fibronectin type III domain‐containing protein 3A) gene includes the fibronectin type III domain. This domain supports it in critical cellular processes, including cell–cell interactions, cell adhesion and signal transduction [16]. Jiang's research targeted the FNDC family. A systematic and in‐depth analysis of the mRNA expression levels of the FNDC family, followed by a prognostic analysis in pan‐cancer, suggests that FNDC3A can become a novel biomarker [17].

Despite the established oncogenic function of SNHG14 in various human cancers, the underlying mechanism in GC remains elusive. This study employed second‐generation HiSeq sequencing and qRT‐PCR analysis to validate the hypothesis of a promising therapeutic target in GC. Our findings suggest that the lncRNA SNHG14 expression is elevated in GC cells, with the upregulation related to improved migration and invasion capabilities. LncRNA mediated this effect by becoming a competitive endogenous RNA, facilitating FNDC3A expression.

## 2 Materials and Methods

### 2.1 Clinical Samples and Ethical Statement

The human gastric cancer and adjacent normal tissues (at least 5 cm away from the tumour edge) were procured from surgical specimens at Gansu Provincial Hospital between September 2020 and September 2024. All the participants provided written informed consent, and the Scientific Research Ethics Committee of Gansu Provincial Hospital approved the study. The fresh tumour tissues were subjected to pathological diagnosis and kept at −80°C. The clinical and pathological characteristics of the patients are depicted in Table 1 and are described following the seventh edition of the American Joint Committee on Cancer (AJCC) staging system.

**TABLE 1:** Relationships between SNHG14 expression and clinicopathological features of GC patients.

Variables		No. of patients	SNHG14 expression		χ^2^	*p*
			High	Low		
Gender			54	28		
	Male	59	38	21	0.196	0.658
	Female	23	16	7		
Age(years)						
	< 60	53	38	15	2.276	0.131
	≥ 60	29	16	13		
Tumour sizes (cm)						
	< 5	35	18	17	5.560	0.017
	≥ 5	47	36	11		
Histologic type						
	Well/Moderate	38	15	23	9.867	0.002
	Poorly	44	39	15		
Tumour depth						
	T1/T2	36	17	19	9.907	0.002
	T3/T4	46	37	9		
Lymph node metastasis						
	Negative	32	16	16	5.866	0.015
	Positive	50	38	12		
Tumour stage						
	I/II	34	21	13	0.441	0.506
	III/IV	48	33	15		

*Note: p* < 0.05 was considered significant.

### 2.2 Cell Culture

The Chinese Academy of Medical Sciences provided the human GC cell lines AGS, MKN‐45, MKN‐28, HGC‐27, NCI‐N87 and the normal human gastric mucosal cell line GES‐1. The NCI‐N87, MKN‐45, MKN‐28, HGC‐27 and GES‐1 cells are maintained in the RPMI‐1640 medium (Gibco, USA). In contrast, the AGS cells are cultured in an F‐12 K medium (Gibco, USA). The media were supplemented using 10% fetal bovine serum (FBS, Gibco, MA, USA), 100 U/mL penicillin and 100 μg/mL streptomycin (Gibco). All the cells were cultured at 37°C in a humidified incubator with 5% CO^2^ based on standard laboratory practice.

### 2.3 RNA Extraction, Reverse‐Transcription PCR and Quantitative Real‐Time PCR (qRT‐PCR)

Total RNA was extracted from cells or tissues with the TRIzol reagent (Invitrogen, MA, USA). Reverse transcription was conducted based on the instructions of the cDNA synthesis kit manufacturer (Takara, Dalian, China). Quantitative real‐time PCR was performed in triplicate to detect mRNA levels with the SYBR Premix Ex Taq II kit (Takara, Dalian, China). The relative abundance of RNA was measured using the standard 2^^(−ΔΔCt)^ method, with GAPDH as the internal control for mRNA and U6 for miRNA. The primer sequences are listed in Table S1.

### 2.4 Cell Transfection

GenePharma (Shanghai, China) designed and synthesised small interfering RNAs (siRNAs) targeting lncRNA/mRNA, microRNA inhibitors and their negative controls. GC cells were plated in 6‐well plates, and transfection was conducted using Lipofectamine 3000 reagent (Invitrogen, USA) after establishing cellular adhesion. The human SNHG14 transcript cDNA and short hairpin RNA (shRNA) targeting SNHG14 were cloned into lentiviral vectors (GenePharma, China) for further use. The stable cells were selected using puromycin, and the sequences are listed in Table S1.

### 2.5 5′ and 3′ Rapid Amplification of cDNA Ends (RACE) Analysis

Total RNA should be extracted with the TriQuick Reagent according to the manufacturer's instructions (Sangon Biotech, China). 5′ and 3′ RACE cDNA libraries are synthesised using the SMARTer RACE 5/3′ Kit (Takara, Beijing, China). The RACE amplification products should be analysed with agarose gel electrophoresis and Sanger sequencing, and the primers are listed in Table S1.

### 2.6 Isolation of Nuclear and Cytoplasmic RNA

A total of 1 × 10^7^ cells were obtained and subjected to subcellular RNA isolation from GC cells with the Cytoplasmic and Nuclear RNA Purification Kit (Beyotime, China). qRT‐PCR helped quantify the SNHG14 expression levels in the cytoplasm and nucleus. U6 was employed as an endogenous control for nuclear RNA. In contrast, GAPDH was used as an endogenous control for cytoplasmic RNA.

### 2.7 RNA Fluorescence In Situ Hybridization (FISH)

A FISH kit from GenePharma Company (Shanghai, China) was used for GC cells. The GenePharma Company (Shanghai, China) designed and synthesised the antisense or sense probes for the SNHG14 linkage sequence. The cells were cultured on glass slides (Merck Millipore) and fixed using 4% paraformaldehyde (Thermo Scientific, Rockford, IL, USA) for 15 min post‐transfection for 24 h. Subsequently, the cells were permeabilised using 0.1% Triton X‐100 at 4°C for 5 min. Then, 100 μL of 2 × SSC was added and incubated for 30 min at 37°C. Later, the cells were incubated using the specific probes overnight at 37°C. After the probe mixture was aspirated, 100 μL of pre‐warmed 0.1% Tween 20 was added to each well at 42°C and washed for 5 min. Then, the cells were washed thrice using 2 × SSC and once with 1 × SSC. Subsequently, a DAPI working solution (Thermo Fisher Scientific) was added to each well and stained for 10–20 min in the dark. After the DAPI working solution was aspirated, the cells were washed twice using phosphate‐buffered saline (PBS) for 5 min each. A confocal microscope was used to capture the images.

### 2.8 Cell Counting Kit‐8 Assay

The cells were seeded into a 96‐well plate at 1 × 10^3^ cellular density per well. CCK‐8 reagent (Apex Bio, USA) was introduced to the wells post‐seeding for 0, 24, 48 and 72 h. Subsequently, the cells were cultured under the aforementioned conditions for another two hours. Subsequently, the absorbance of each well was determined at 450 nm with a microplate reader (Thermo Fisher Scientific Inc.).

### 2.9 5‐Ethynyl‐2′‐Deoxyuridine (Edu) Assay

The EdU Cell Proliferation Detection Kit (Beyotime, China) helped assess cell proliferation. From each experimental group, tumour cells (2 × 10^4^ cells per well) were seeded inside a 24‐well plate and incubated for 24 h. Later, the cells were incubated within a medium with 50 mM EdU for two more hours. The cells were incubated using Hoechst 33,342 for 30 min to stain the DNA, followed by observing them using an inverted fluorescence microscope (Olympus IX73, Japan). Five random fields were captured in each experiment using the EdU assay. The images were subsequently processed and analysed using the ImageJ 1.53 software. The incorporation rate of the thymidine analog 5‐ethynyl‐2′‐deoxyuridine (EdU) was determined as the ratio of EdU‐positive cells to the number of cells positive for the nuclear dye Hoechst 33,342 within each field of view.

### 2.10 Colony Formation

1 × 10^3^ transfected cells were plated within a 6‐well plate and cultured for two weeks under the abovementioned conditions. The culture medium must be changed on a tri‐weekly basis. Then, the colonies were fixed using methanol for 20 min and stained with a 0.1% crystal violet solution for another 20 min. After staining, the cells were washed thrice using PBS, photographed and visualised under a light microscope.

### 2.11 Transwell Assay

For invasion assays, transwell chambers (24‐well plates; 8‐μm pore size, Corning Costar Corp.) were coated using Matrigel (BD Biosciences, Bedford, MA, USA). However, they were left uncoated for migration assays. A 200 μL serum‐free medium containing the transfected cells (6 × 10^4^ cells per well) was introduced to the upper chamber. In contrast, 600 μL of medium containing 10% FBS was added to the lower chamber. After a 24‐h incubation period, non‐migrated cells were removed from the upper insert surface with a cotton swab. The cells that had migrated via the permeable membrane were fixed in methanol, stained using crystal violet and counted under a light microscope (Olympus Corporation, Tokyo, Japan) at 20x magnification within random fields of all the wells.

### 2.12 Wound Healing Assay

The transfected cells were seeded inside a 6‐well plate at a density of 2 × 10^5^ cells per well and cultured until achieving 80% confluence, based on standard practice. Then, the cells were gently scraped vertically using a 200 μL pipette tip and washed thrice with PBS. Subsequently, the cells were cultured within a serum‐free medium. The migration was documented under an inverted microscope at the initial (0 h) and the final (48 h) time points. The degree of mobility was determined with the following formula: Mobility = ((Wound length at 0 h − Wound length at 48 h)/Wound length at 0 h) × 100%.

### 2.13 IF (Immunofluorescence)

The transfected cells were fixed using 4% formaldehyde for 15 min and washed with PBS. The immobilised cells were treated with pepsin, dehydrated using ethanol and permeabilised for 20 min in Triton X‐100 (Sigma‐Aldrich). The cells were blocked with goat serum and incubated with the first antibody (Proteintech, China) overnight at 4°C. Later, they were incubated using the appropriate rhodamine conjugate for one hour. Subsequently, DAPI (Invitrogen) helped wash and incubate the cells DAPI for nuclear staining. Cellular immunofluorescence was observed with a fluorescence microscope (DMI4000B, Leica).

### 2.14 Flow Cytometry Analysis

The cell cycle of gastric cancer cells was analysed with flow cytometry. After a 48‐h transfection period, the cells were fixed in 75% ethanol for 30 min and stained using propidium iodide (Sigma, Missouri, USA) for 15 min in the dark. The apoptotic assay utilised an Annexin V‐APC/PI Apoptosis Detection Kit (Multisciences, China). The early to late apoptotic cell ratio helped measure the apoptotic rate. Then, the cell cycle and apoptotic assay were evaluated using a BD Biosciences FACSCalibur flow cytometer (BD Biosciences, USA).

### 2.15 Western Blotting

The transfected cells were harvested and lysed on ice using RIPA lysis buffer. The buffer possesses a cocktail of protease inhibitors and different phosphatase inhibitors. The protein concentration was determined with the bicinchoninic acid (BCA) protein assay (Thermo Fisher Scientific, Waltham, MA). The protein lysates were subjected to SDS‐PAGE on a 10% gel and transferred onto a PVDF membrane (Millipore). Then, the lysates were blocked using 5% non‐fat milk for two hours. The primary antibodies, namely, cleaved‐caspase 3, Bcl‐2, Bax, E‐cadherin, N‐cadherin, vimentin, FNDC3A, ZNF460 and GAPDH, were procured from Proteintech (Wuhan, China). Horseradish peroxidase (HRP)‐conjugated anti‐rabbit antibodies were the secondary antibodies used. The membranes were incubated using the corresponding primary and secondary antibodies at 4°C overnight, providing gentle agitation. Later, protein levels were quantified using an HRP‐labelled secondary antibody and an ECL chemiluminescence detection system across a Bio‐Rad ChemiDoc XRS system (Bio‐Rad, USA).

### 2.16 Dual Luciferase Assays

The miR‐206 binding sites were cloned with predictive sequences and their mutant SNHG14 and FNDC3A fragment sequences into the pmirGLO vector (Gene Pharma, USA) for subsequent analysis. These wild‐type and mutant vectors were sequenced and designated SNHG14‐Wt, SNHG14‐Mut, FNDC3A‐Wt, FNDC3A‐Mut, SNHG14 promoter‐Wt and SNHG14 promoter‐Mut. The cells were seeded at a density of 1 × 10^4^ cells per well inside a 96‐well plate and co‐transfected with different treatments using Lipofectamine 3000 to obtain 75% confluence. After 48 h post‐transfection, the relative luciferase activity was quantified using the Dual‐Luciferase Reporter Assay System (Promega, USA) based on the manufacturer's instructions. The cells were seeded at a density of 1 × 10^4^ cells per well within a 96‐well plate and transfected through different treatments using Lipofectamine 2000, providing a 75% confluence. After 48 h post‐transfection, relative luciferase activity was determined. This was performed after 48 h of transfection, with firefly luciferase activity normalised to Renilla luciferase activity.

### 2.17 High‐Throughput Sequencing and Target Gene Prediction

The RNA microarray analysis was performed to identify differentially expressed genes (DEGs) and microRNAs (DEMs) in gastric cancer (GC) cells with or without SNHG1414 knockdown. OE Biotech Co. Ltd. (Shanghai, China) helped undergo microarray RNA sequencing using the Illumina HiSeq X Ten platform. Differential expression analysis was conducted using the DESeq2 software, wherein genes satisfying the *q*‐value < 0.05 and fold change > 2 or fold change < 0.5 criteria were defined as DEGs or DEMs. Hierarchical clustering analysis of the DEGs was conducted with R (version 3.2.0) to demonstrate the expression patterns of genes among different groups and samples. The R package ‘ggradar’ plotted a radar chart of the top 30 genes to demonstrate the expression changes involving upregulated or downregulated genes. Subsequently, the study performed a hypergeometric distribution algorithm‐based enrichment analysis for GO, KEGG, Pathway, Reactome and WikiPathways to filter significantly enriched functional terms. R (version 3.2.0) helped plot bar charts, chord diagrams, or enrichment analysis circle charts for the significantly enriched functional terms.

The downstream miRNAs controlled by SNHG14 and their potential binding sites were predicted with the LncRNA2Target database (http://bio‐computing.hrbmu.edu.cn/lncrna2target/). The predicted target genes along with the potential binding sites of miR‐206 from Starbase (http://starbase.sysu.edu.cn/), miR‐Target (https://mirtarbase.cuhk.edu.cn/~miRTarBase/), miRDB (https://www.mirdb.org) and TargetScan (https://www.targetscan.org) helped search miRNA targets. Cytoscape 3.9.1 generated the ncRNA‐miRNA‐mRNA regulatory network.

### 2.18 Immunohistochemistry (IHC)

The tissue was prepared to embed in paraffin wax and cut into sections of 4 mm in thickness. These sections were transferred onto glass slides. Then, the tissue was deparaffinised using xylene and rehydrated with a graded ethanol series. Subsequently, the specimens were incubated overnight with the primary antibody, followed by HRP‐conjugated secondary antibody (ZSGB‐Bio, Beijing, China) incubation. Subsequently, the specimens were detected using 3,3′‐diaminobenzidine and haematoxylin.

### 2.19 RNA Immunoprecipitation (RIP) Assay

The Magna RIP RNA‐Binding Protein Immunoprecipitation Kit (Millipore, Billerica, MA, USA) helped conduct RIP assays. The RIP immunoprecipitation buffer comprised magnetic beads conjugated using a human anti‐Argonaute 2 (AGO2) antibody (Millipore, Billerica, MA, USA) or mouse IgG (Millipore, Billerica, MA, USA) as a negative control. The GC cells were lysed in a complete RNA lysis buffer. Subsequently, the resulting cell lysates were incubated using the RIP immunoprecipitation buffer. The complexes were washed three times with cold PBS, and qRT‐PCR helped quantify the enrichment of precipitated SNHG14 and FNDC3A.

### 2.20 RNA Pull‐Down Assay

Based on the instructions provided by the manufacturer of the RNA pull‐down kit (Bersinbio, China), biotin‐labelled miR‐206 was transcribed and marked in vitro. The housekeeping gene β‐actin became the control. After establishing the secondary structure of the biotin‐labelled miR‐206 (probe), the probe was incubated using streptavidin‐coated magnetic beads for two hours, producing probe‐bound magnetic beads. Subsequently, the cell lysates were incubated using the probe‐bound magnetic beads for two hours. Later, qRT‐PCR helped analyse the SNHG14 and FNDC3A transcripts bound to miR‐206.

### 2.21 Chromatin Immunoprecipitation (ChIP) Assay

Chromatin immunoprecipitation (ChIP) analysis was conducted based on the instructions of the manufacturer, using the EZ‐ChIP Chromatin Immunoprecipitation Kit (Millipore, MA, USA). Immunoprecipitation was conducted with antibodies specific to ZNF460 (Proteintech, China) and ICAM1 (Proteintech, China). Quantitative ChIP‐derived DNA analysis was performed using quantitative reverse transcription qRT‐PCR reactions. The primer sequences are listed in Table S1.

### 2.22 In Vivo Assay

The BALB/c nude mice (4–6 weeks old, with nearly 20 g weight) were obtained from the Laboratory Animal Science Center of Lanzhou University (Gansu, China) and housed inside a specific pathogen‐free facility. Around 10 BALB/c nude mice were used in the study, randomly assigned with a computer‐generated random number list. A suspension of cells transfected with sh‐NC or shSNHG14‐1 (5 × 10^6^) in 0.1 mL PBS was inoculated within the right flank of the mice to establish in vivo tumour growth or metastasis models. Tumour size was measured weekly. After 28 days post‐inoculation, the mice were euthanised, followed by excising and measuring the tumours. The determination was based on the ratio of positively stained cells to the number of cells across three randomly selected areas. This was assessed by immunostaining using Ki‐67, E‐cadherin, N‐cadherin, Vimentin and FNDC3A. All the experiments involving animals were conducted following the guidelines provided by the Lanzhou University Animal Care and Use Committee.

### 2.23 Statistical Analysis

Each experiment was conducted on three occasions, and the data highlighted represent one such experiment. The data were analysed with GraphPad Prism 9.0 and the R programming language (version 4.1.3). The statistical tests were validated appropriately for the data set in question. An analysis of variance (ANOVA) was performed after satisfying the requisite assumptions. The mean standard deviation (SD) represents the obtained results. The significance of the observed differences was evaluated using Student's t‐tests and one‐way ANOVA, with the latter performed using SPSS 16.0 software. An overall survival analysis was determined using the Kaplan–Meier method. A *p*‐value < 0.05 was considered statistically significant.

## 3 Results

### 3.1 Identification and Characterisation of lncRNA SNHG14 in GC

**FIGURE 1:**
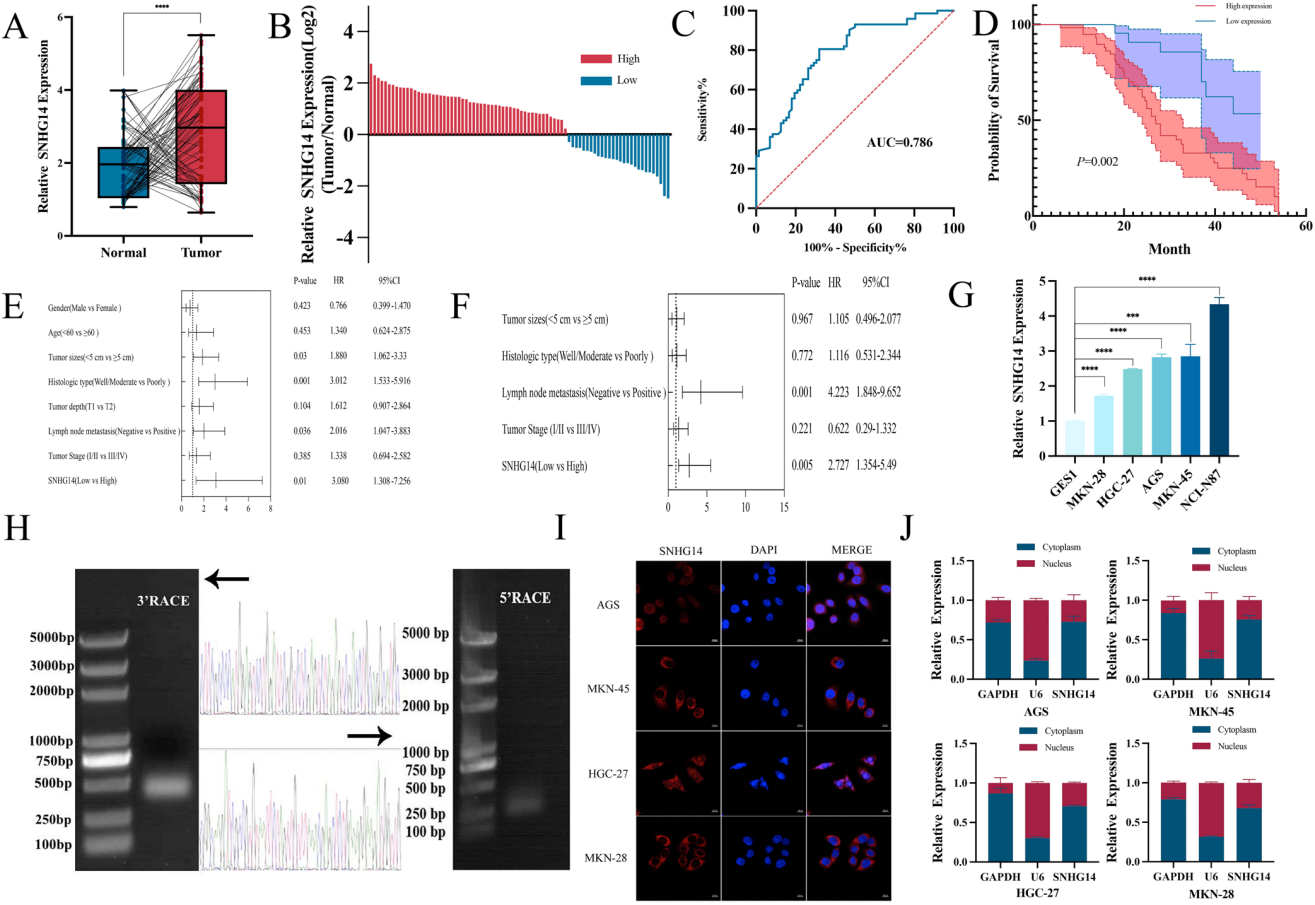
Identification and characterisation of lncRNA SNHG14 in GC. (A, B) qRT‐PCR analysis shows the expression of SNHG14 in 82 pairs of GC and adjacent normal gastric tissues. (C) ROC curve for SNHG14 expression in normal and gastric cancer tissues. (D) Kaplan–Meier survival curve of overall survival in 82 GC patients according to the lncRNA SNHG14 expression. (E, F) Univariate regression and multivariate survival method (Overall Survival) of prognostic covariates in 82 GC patients. (G) The level of SNHG14 in five GC cell lines and the normal gastric epithelial cells was examined by qRT‐PCR. (H) Representative images of PCR products from 3′ RACE and 5′ RACE. (I, J) RNA FISH and nuclear‐cytoplasmic fractionation assays showed subcellular localization of SNHG14 in AGS, MKN‐45, HGC‐27 and MKN‐28 cells. *p < 0.05, **p < 0.01 and ***p < 0.001.

To investigate the underlying role of lncRNA SNHG14 in GC progression, qRT‐PCR was conducted to assess the SNHG14 expression in 82 pairs of GC tissues retrieved from the Gansu Provincial People's Hospital. SNHG14 was highly expressed in GC tissues compared to adjacent (tissues (Figure 1A,B). To assess the diagnostic significance of SNHG14, the receiver operating characteristic (ROC) curve was plotted using the SNHG14 expression values in GC tissues and adjacent control tissues. The area under the ROC curve (AUC) for SNHG14 was 0.786, suggesting that SNHG14 has a favourable diagnostic efficacy for GC and the potential to serve as a diagnostic marker (Figure 1C). The clinical and pathological characteristics of GC patients depicted that high SNHG14 expression was significantly related to tumour size (≥ 5 cm), polypoid histologic type, tumour depth (T3/T4) and positive lymph node metastasis (Table 1). The survival analysis results indicated that patients having high SNHG14 expression showed significantly poorer overall survival rates (Figure 1D). Univariate and multivariate Cox regression analysis highlighted that lncRNA SNHG14 expression (*p* = 0.015) and positive lymph node metastasis (*p* = 0.03) were independent prognostic factors for GC patients (Figure 1E,F). Additionally, SNHG14 expression was notably elevated in GC cells compared to normal gastric epithelial cells (Figure 1G).

SNHG14 is on human chromosome 15q11.2, and the LNCipedia database provides five annotated SNHG14 transcripts (Figure S1A,B). The ORFfinder tool on PubMed reveals no open reading frame (ORF) on the positive (+) strand longer than 150 nucleotides in the SNHG14 sequence, indicating a non‐coding RNA (Figure S1C). The 5′ and 3′ RACE experiment results identified a 489 bp transcript as the predominant isoform of SNHG14 (Figure 1H). The complete sequence of SNHG14 is presented in Figure S1D. Moreover, FISH and nuclear‐cytoplasmic fractionation assays conducted on gastric cancer cells indicate that SNHG14 is predominantly localised in the cytoplasm, suggesting that it may exert its oncogenic effects in gastric cancer through post‐translational modifications (Figure 1I,J).

### 3.2 Altering the Expression of SNHG14 Affects the Proliferation, Migration and Invasion of GC Cells

**FIGURE 2:**
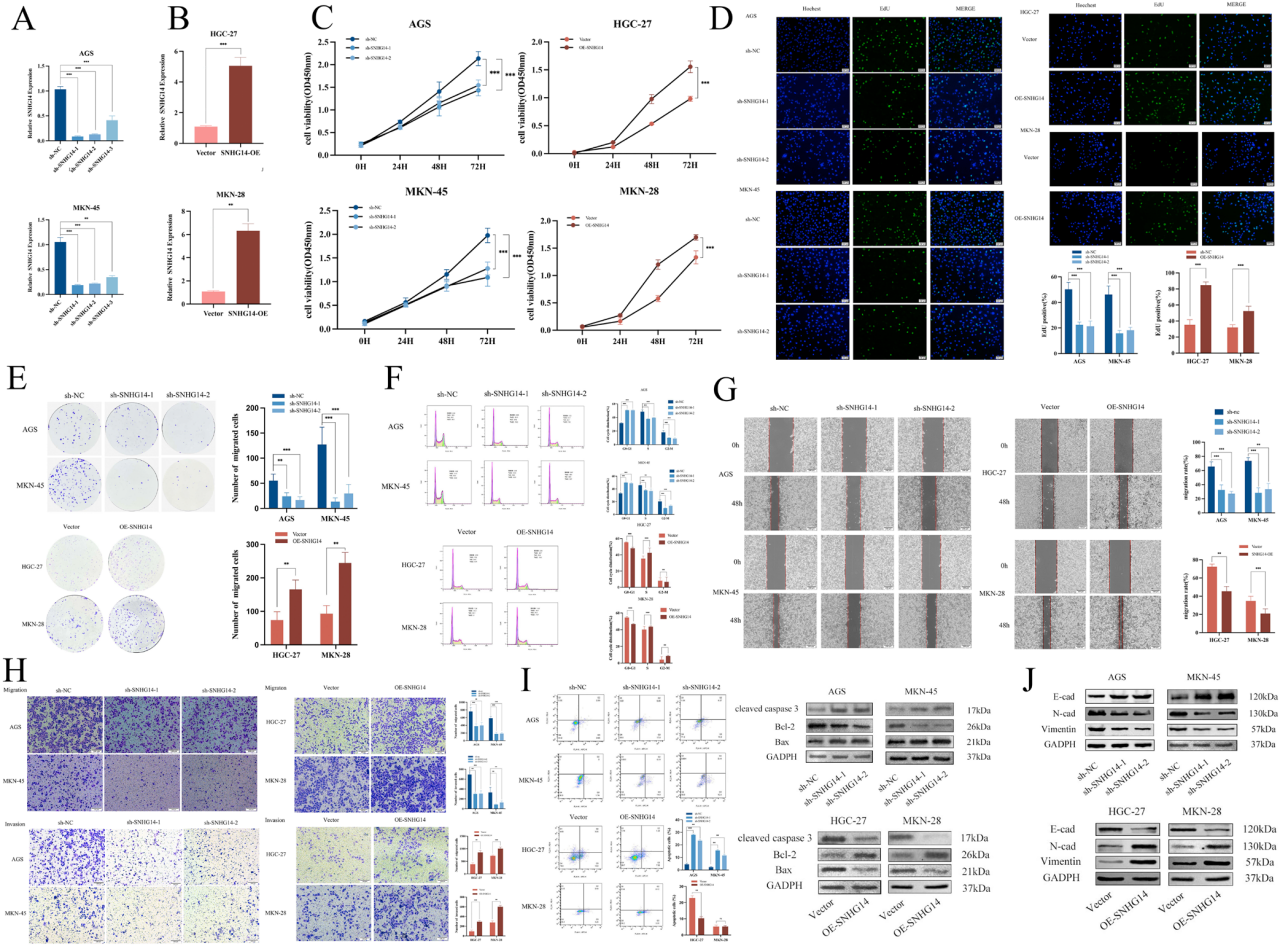
Altering the expression of SNHG14 affects the proliferation, migration and invasion of GC cells. (A, B) The transfection efficiency in GC cells after transfections was confirmed by qRT‐PCR. (C, D, E, F) The proliferation of indicated CRC cells was evaluated by CCK‐8 assay, EDU assay, colony formation assay and flow cytometry analysis. (G, H) The effect of SNHG14 on GC cell migration and invasion was tested by wound healing assay and transwell assay. (I) The impact of SNHG14 on apoptosis was tested by flow cytometry and western blotting. (J) The expression of EMT‐related proteins was detected through conducting western blot. *p < 0.05, **p < 0.01 and ***p < 0.001.

To elucidate the function of SNHG14 in GC proliferation and metastasis in vitro, we knocked down the SNHG14 expression by stably transfecting AGS and MKN‐45 cells with shRNA. We elevated SNHG14 expression by transfecting the overexpression plasmid OE‐SNHG14 into HGC‐27 and MKN‐28 cells (Figure 2A,B). Value addition was assessed using CCK‐8, EDU fluorescence staining, a clone formation assay and flow cytometry. The results indicated that the proliferative capacity of GC cells was diminished after SNHG14 knockdown. In contrast, the proliferative capacity of GC cells was improved after SNHG14 overexpression (Figure 2C–F). Secondly, the impact of SNHG14 on GC metastasis was assessed using wound healing and Transwell assays. The results indicated that the metastatic capacity of GC cells was reduced after SNHG14 knockdown, whereas its overexpression enhanced the metastatic ability (Figure 2G,H). Flow cytometry and Western blotting helped detect the impact of SNHG14 on the apoptosis of GC cells. Although flow cytometry did not detect any significant differences in MKN‐28 cells, there was a marked discrepancy in the expression of proteins associated with apoptosis (*p* = 0.912). The results demonstrated that the apoptotic ability of GC cells was elevated after the SNHG14 knockdown, while its overexpression weakened the apoptotic capability (Figure 2I). Additionally, the protein expression associated with epithelial‐mesenchymal transition (EMT) was examined. Upon SNHG14 knockdown, E‐cadherin expression was increased with a concomitant decline in N‐cadherin and vimentin expression. However, the trend was the opposite in the protein levels of the EMT‐related genes upon SNHG14 overexpression (Figure 2J). Furthermore, the immunofluorescence staining results for the above three molecules are based on the WB findings (Figure S2). Therefore, SNHG14 plays a significant role in the proliferation, migration and invasion of GC cells, functioning as a pro‐carcinogenic factor and affecting the proliferation and metastasis of GC.

### 3.3 Combined miRNA Sequencing and Transcriptome Sequencing Analysis Determines SNHG14 Downstream Mechanisms

**FIGURE 3:**
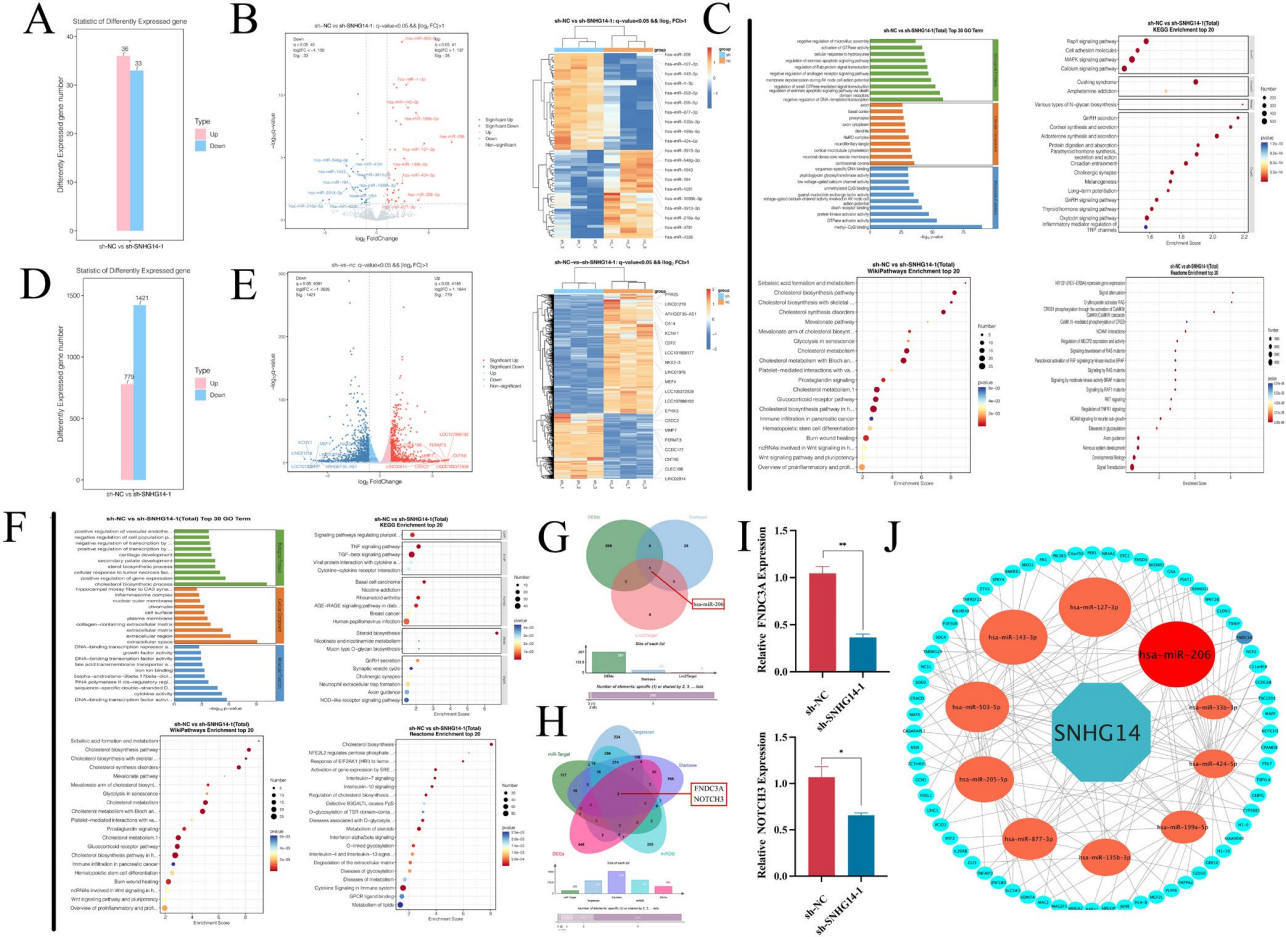
Combined miRNA sequencing and transcriptome sequencing analysis predicts SNHG14 downstream mechanisms. (A) Differentially expressed miRNAs in sh‐NC and sh‐SNHG14‐1 groups. (B) Top 10 differential miRNAs shown in volcano plots and heatmaps. (C) GO, KEGG, WikiPathways and Reactome enrichment analysis in DEMs. (D) Differentially expressed mRNAs in sh‐NC and sh‐SNHG14‐1 groups. (E) Top 10 differential mRNAs shown in volcano plots and heatmaps. (F) GO, KEGG, WikiPathways and Reactome enrichment analysis in DEGs. **(**G) The intersection of different online databases and DEMs was presented by a Venn graph. (H) The intersection of different online databases and DEGs was presented by a Venn graph. **(**I) The expression of FNDC3A and NOTCH3 in GC cells under transfections was examined by qRT‐PCR. (J) Differentially expressed gene interaction network analysis. *p < 0.05, **p < 0.01 and ***p < 0.001.

A microRNA (miRNA) microarray analysis identified differentially expressed miRNAs and their functions between the sh‐NC and sh‐SNHG14‐1 groups in GC patients. The study identified 69 differentially expressed miRNAs, including 36 upregulated and 33 downregulated genes (Figure 3A). The top 10 differential miRNAs in each group were demonstrated using volcano plots and heatmaps (Figure 3B). The Gene Ontology (GO) enrichment analysis suggested that the differential genes were primarily associated with biological processes such as cholesterol biosynthesis, cellular components like the extracellular space, and molecular functions mainly linked with DNA‐binding transcription factor activity. Kyoto Encyclopedia of Genes and Genomes (KEGG) and WikiPathways enrichment analyses highlighted the predominant enrichment of differential genes in the MAPK signalling pathway. The reactome enrichment analysis showed enrichment in signal transduction processes (Figure 3C).

mRNA sequencing identified 2200 differentially expressed genes between the sh‐NC and sh‐SNHG14‐1 groups, involving 779 upregulated and 1421 downregulated genes (Figure 3D). The top 10 differential miRNAs in each group were highlighted within volcano plots and heatmaps (Figure 3E). The GO enrichment analysis suggested that the differential genes were primarily associated with the biological process of negative regulation of DNA‐templated transcription. The study also included cellular components such as the centrosome corona and molecular functions predominantly linked to methyl‐CpG binding. The KEGG enrichment analysis depicted that the differential genes were primarily enriched within the TGF‐β signalling pathway. In contrast, the Reactome enrichment analysis depicted results associated with Cytokine Signalling in the Immune System. The WikiPathways enrichment analysis highlighted that the differential genes were predominantly related to the cholesterol biosynthesis pathway in hepatocytes (Figure 3F).

A joint miRNA and mRNA sequencing analysis was conducted to ascertain the specific targets of SNHG14. An initial intersection was performed between the upregulated DEMs and the Starbase database LncRNA2Target, selecting miR‐206 that targets the sponge binding of SNHG14 (Figure 3G). To identify potential miR‐206 targets, the top 250 differentially expressed genes (DEGs) were filtered using Starbase, miR‐Target, miRDB and Targetscan databases for identifying the common genes (Figure 3H). The results demonstrated that FNDC3A and NOTCH3 were identified as common genes among the five databases. qRT‐PCR helped detect the expression levels of FNDC3A and NOTCH3 in the sh‐NC and sh‐SNHG14‐1 groups to characterise FNDC3A as the miRNA targeting the sponge binding of miR‐206 (Figure 3I). Therefore, it was hypothesised that SNHG14 regulates FNDC3A by targeting the miR‐206 sponge binding via a ceRNA mechanism (Figure 3J).

### 3.4 SNHG14 Acts as a Molecular Sponge for miR‐206 in GC Cells

**FIGURE 4:**
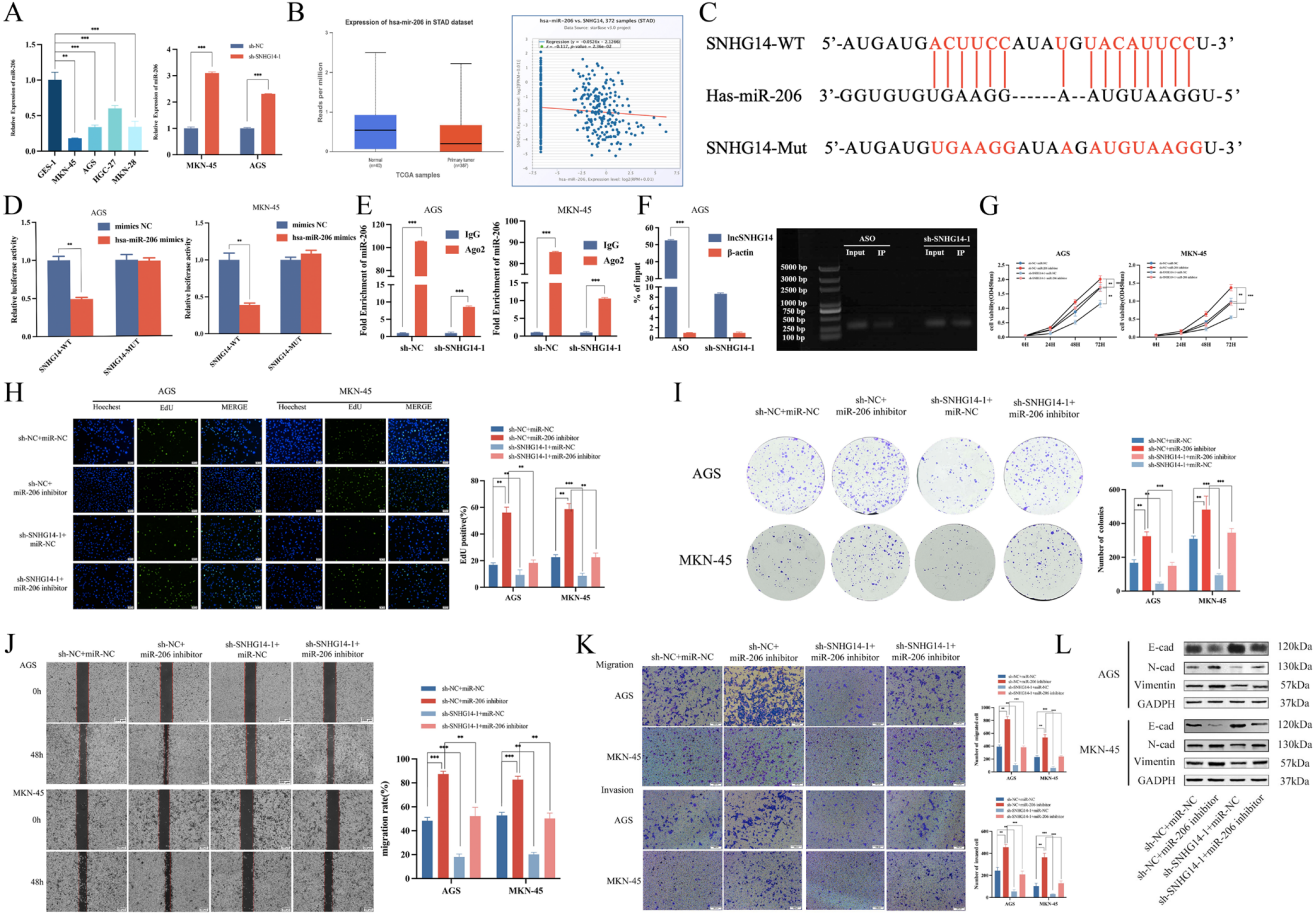
SNHG14 acts as a molecular sponge for miR‐206 in GC cells. (A) The expression of miR‐206 in GC cell lines and GC cells under different transfections was examined by qRT‐PCR. (B) The expression of miR‐206 in GC and adjacent normal gastric tissues and the relationship of the expression of SNHG14 in the starbase database. (C) Bioinformatics methods were employed to identify potential binding sites between SNHG14 and miR‐206. D Luciferase reporter assay was conducted to assess the influence of SNHG14 on miR‐206. **(**E) RIP assay was performed to determine the association between SNHG14 and miR‐206. **(**F**)** RNA pull‐down assay showing the miR‐206 binding with biotinylated SNHG14. (G, H, I) CCK8, EdU and colony formation assays show the proliferation rates of co‐transfected AGS and MKN‐45 cells with sh‐SNHG14 and miR‐206 inhibitors. (J, K) The wound healing assay and transwell assay show the metastasis of co‐transfected AGS and MKN‐45 cells with sh‐SNHG14‐1 and miR‐206 inhibitor. (L) The expression of EMT‐related proteins was detected in co‐transfected AGS and MKN‐45 cells with sh‐SNHG14 and miR‐206 inhibitors. **p < 0.05, **p < 0.01 and ***p < 0.001.

Given the preceding miRNA sequencing and public database analysis, miR‐206 has a high probability of binding to the SNHG14 sponge. The initial examination of miR‐206 expression in GC cells indicated that miR‐206 is underexpressed in these cells (Figure 4A). Moreover, the miR‐206 expression increased upon silencing SNHG14. Searching the StarBase and UNLCAN databases revealed that miR‐206 is underexpressed in GC patients and inversely correlates with SNHG14 expression (Figure 4B). Bioinformatics methods helped identify potential binding sites between SNHG14 and miR‐206 (Figure 4C). Dual‐luciferase reporter gene assays indicated that miR‐206 significantly diminished the relative luciferase activity of the wild‐type SNHG14 3′UTR (Figure 4D). Furthermore, a RIP assay was performed using an AGO2 antibody. After the SNHG14 knockdown, the enrichment level of miR‐206 in the sh‐SNHG14‐1 group was significantly decreased compared to the sh‐NC group (Figure 4E). The RNA pull‐down assay consistently validated the interaction between SNHG14 and miR‐206. After the SNHG14 knockdown, SNHG14 enriched by the miR‐206 probe was significantly reduced compared to the control group (Figure 4F). We conducted a co‐transfection experiment in AGS and MKN‐45 cells using sh‐SNHG14 and miR‐206 inhibitors to elucidate the biological interaction between SNHG14 and miR‐206 in GC. The proliferative capacity was determined using CCK‐8 assays, EdU assays and colony formation assays (Figure 4G–I). The migratory ability was evaluated by utilising Transwell migration and invasion assays, other than wound healing assays (Figure 4J,K). Furthermore, the EMT marker expression was determined using Western blot analysis (Figure 4L). The results of the experimental investigation suggest that the miR‐206 inhibitor can reverse the promoting effects of SNHG14 on GC cell proliferation, migration and EMT. Our findings depict that SNHG14 targets and sponges miR‐206 via a ceRNA mechanism, impacting the progression of GC cells.

### 3.5 miR‐206 Suppresses Cell Proliferation and Migration Capacity by Targeting FNDC3A in Gastric Cancer

**FIGURE 5:**
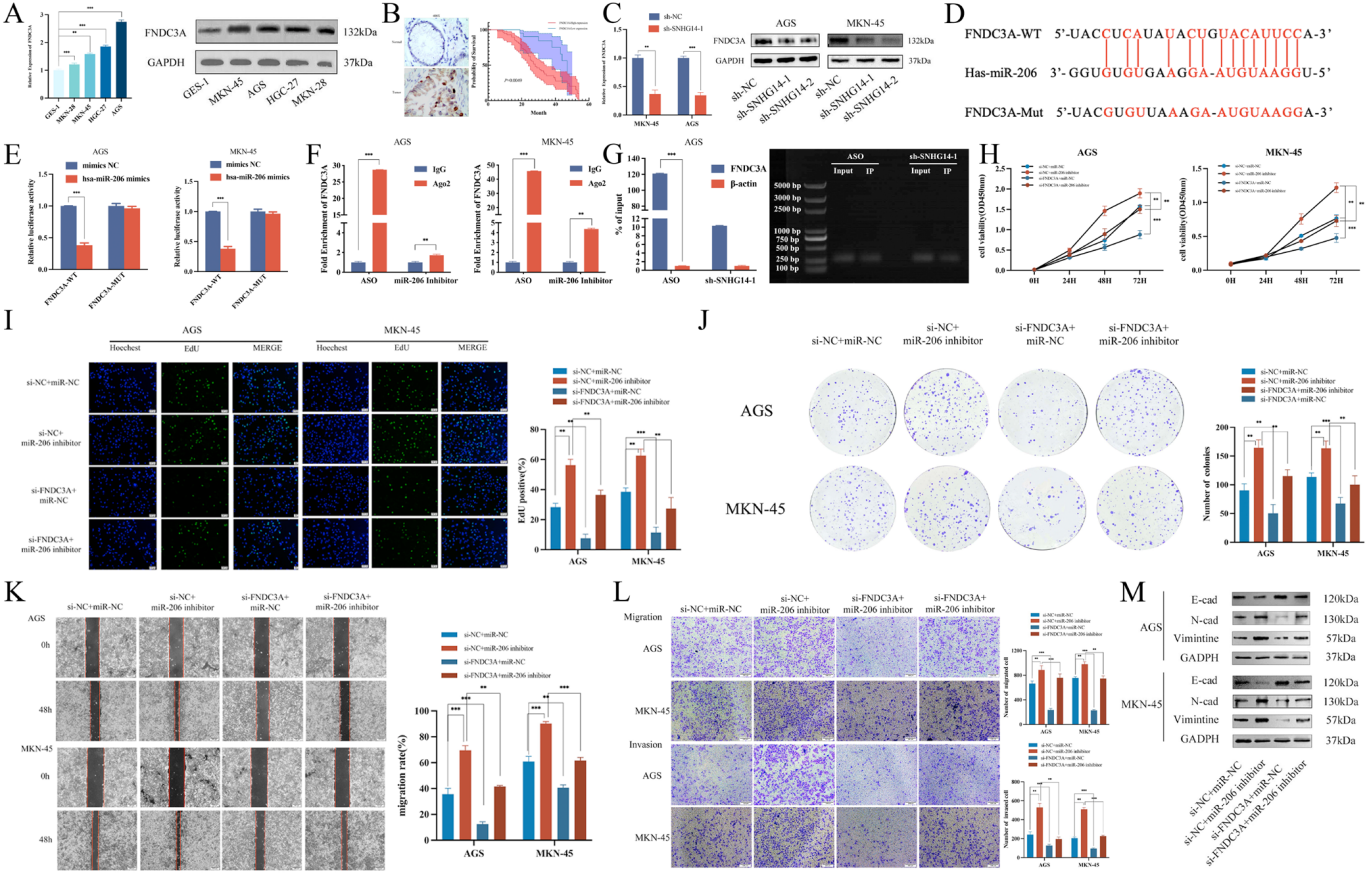
miR‐206 suppresses cell proliferation and migration capacity by targeting FNDC3A in gastric cancer. (A) The expression of FNDC3A in GC cell lines. (B**)** Expression of FNDC3A in GC and adjacent tissues and Kaplan–Meier analysis of the correlation between the expression of FNDC3A and OS in GC patients. (C) GC cells under different transfections were examined by qRT‐PCR and western blot. (D) Bioinformatics methods were employed to identify potential binding sites between miR‐206 and FNDC3A. (E) Luciferase reporter assay was conducted to assess the influence of miR‐206 and FNDC3A. (F) RIP assay was performed to determine the association between miR‐206 and FNDC3A. (G) RNA pull‐down assay showing the miR‐206 binding with biotinylated FNDC3A. **(**H, I, J) CCK8, EdU and colony formation assays show the proliferation rates of co‐transfected AGS and MKN‐45 cells with si‐FNDC3A and miR‐206 inhibitors. (K, L) The wound healing assay and transwell assay show the metastasis of co‐transfected AGS and MKN‐45 cells with s si‐FNDC3A and miR‐206 inhibitor. (M) The expression of EMT‐related proteins was detected in co‐transfected AGS and MKN‐45 cells with si‐FNDC3A and miR‐206 inhibitor. *p < 0.05, **p < 0.01 and ***p < 0.001.

A joint analysis of mRNA sequencing results and online databases provided the hypothesis that FNDC3A could serve as a miR‐206 target. This hypothesis is substantiated by initially evaluating FNDC3A expression in GC cells using qRT‐PCR, western blot analysis and online databases. FNDC3A demonstrated elevated levels of expression in gastric cancer cells and tissues, with this heightened expression correlating to poor prognosis (Figure 5A,B). Moreover, the expression of FNDC3A decreased following the silencing of SNHG14 (Figure 5C). Bioinformatics methods helped identify potential binding sites between FNDC3A and miR‐206 (Figure 5D). A co‐transfection of hsa‐miR‐206 and FNDC3A‐WT plasmids was performed for 48 h to validate the direct interaction between miR‐206 and FNDC3A. Compared to the control group, the luciferase expression was reduced, depicting that hsa‐miR‐206 binds to the target site of FNDC3A (Figure 5E). Furthermore, RIP assays demonstrated that the FNDC3A enrichment level was significantly diminished in the miR‐206 inhibitor group relative to the ASO group after the miR‐206 knockdown (Figure 5F). The RNA pull‐down assay consistently verified the interaction between miR‐206 and FNDC3A. The FNDC3A level enriched by the miR‐206 probe was significantly diminished compared to the control group (Figure 5G). Subsequently, CCK‐8 assays, colony formation assays and EdU assays helped evaluate the alterations in the proliferative capacity of GC cells post‐co‐transfection (Figure 5H–J). The migratory capacity of the cells was assessed using transwell migration, invasion and wound healing assays (Figure 5K,L). Additionally, the EMT changes were evaluated by detecting EMT markers using WB (Figure 5M). Our experimental procedures demonstrate that FNDC3A silencing can restore the tumour‐suppressive effects of miR‐206 on cell proliferation, migration and EMT. Our findings highlight that the oncogenic function of FNDC3A is partly dependent on its ability to negatively mediate miR‐206.

### 3.6 ZNF460 Transcriptionally Represses SNHG14

**FIGURE 6:**
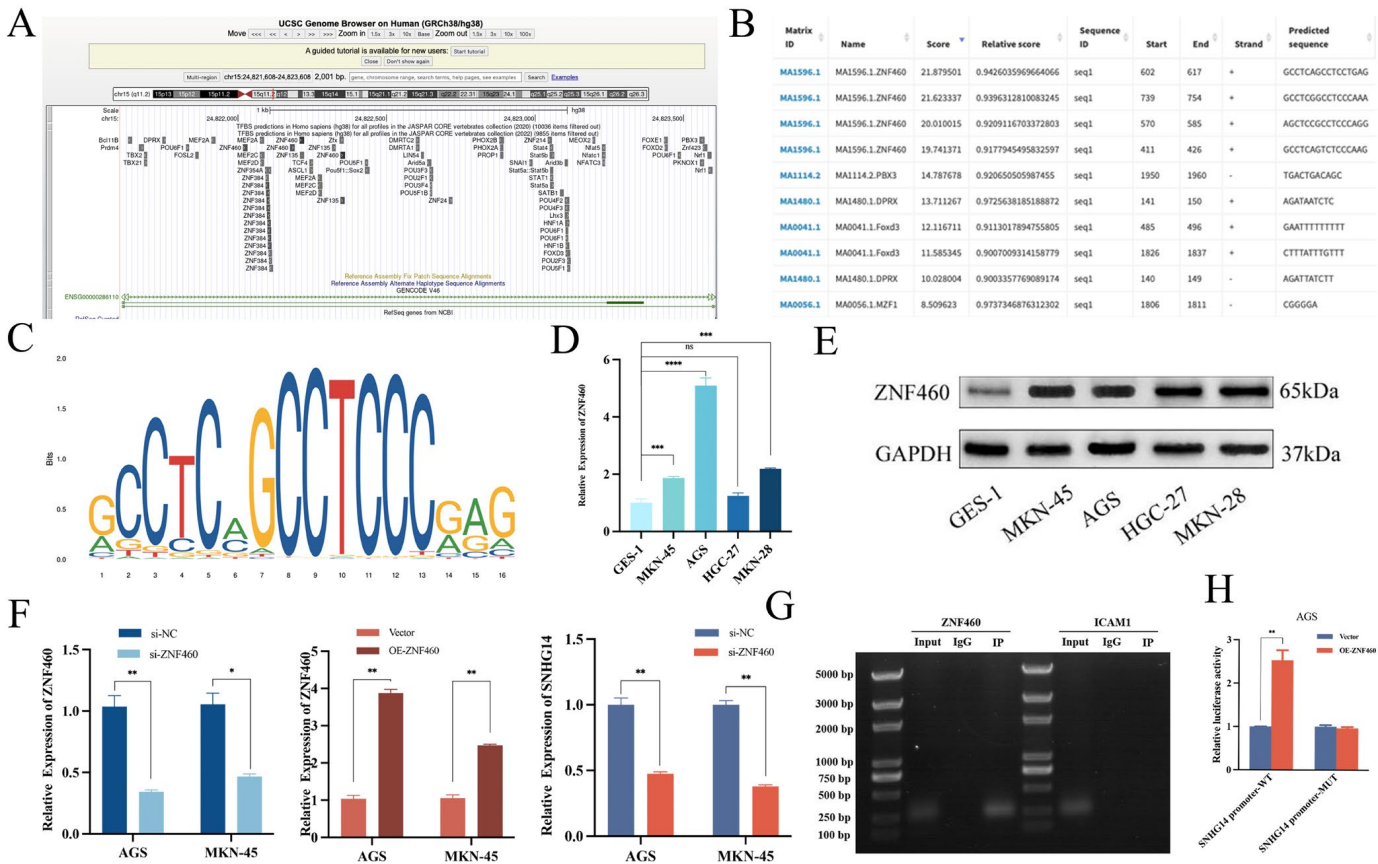
SNHG14 is transcriptionally repressed by ZNF460. (A, B, C) Potential transcription factors of the SNHG14 promoter were projected through linking the UCSC database with the JASPAR website. (D, E) The expression of ZNF460 in GC cell lines was examined by qRT‐PCR and Western blot. (F) The transfection efficiency in GC cells after transfections was confirmed by qRT‐PCR, and SNHG14 expression was diminished after the transfection of specific si‐ZNF460 vectors. (G) ChIP assay was performed to determine the association between ZNF460 and SNNHG14 promoter. (H) Luciferase reporter assays were performed to confirm the binding relationship between ZNF460 and SNNHG14 promoter in AGS cells. *p < 0.05, **p < 0.01 and ***p < 0.001.

Evidence suggests that several key transcription factors play a role in the dysregulation of lncRNAs in human cancer cells [18]. Subsequently, the upstream transcription factors of SNHG14 were investigated. The utilisation of bioinformatics tools, particularly the UCSC Genome Browser (http://genome.ucsc.edu) and JASPAR (http://jaspar.genereg.net) helped identify a single binding site for ZNF460 on the SNHG14 promoter (Figure 6A–C). First, the ZNF460 protein expression was confirmed in GC cell lines with qRT‐PCR and western blot analysis. These results highlighted that the ZNF460 expression level was significantly higher in GC cell lines than in the normal gastric epithelial cells (Figure 6D,E). Subsequently, the transfection efficiency in GC cells was confirmed by qRT‐PCR, and SNHG14 expression was diminished in AGS and MKN‐45 after the transfection with specific si‐ZNF460 vectors (Figure 6F). CHIP assays were performed with a ZNF460 antibody, followed by qRT‐PCR detection using SNHG14 promoter‐specific primers. The results highlighted the presence of the target band in the input and immunoprecipitation (IP) groups (Figure 6G). This suggested that ZNF460 can bind to the LncSNHG14 promoter region. Subsequent dual‐luciferase reporter gene assays highlighted that ZNF460 overexpression enhanced luciferase activity when co‐transfected with the SNHG14 promoter‐WT. In contrast, no significant change could be observed in the luciferase activity of the SNHG14 promoter‐Mut (Figure 6H). The study results show that ZNF460 may be necessary for the SNHG14 upregulation within GC cells.

### 3.7 Depletion of SNHG14 Inhibits Tumour Growth and Metastasis in Nude Mouse Cell Xenografts

**FIGURE 7:**
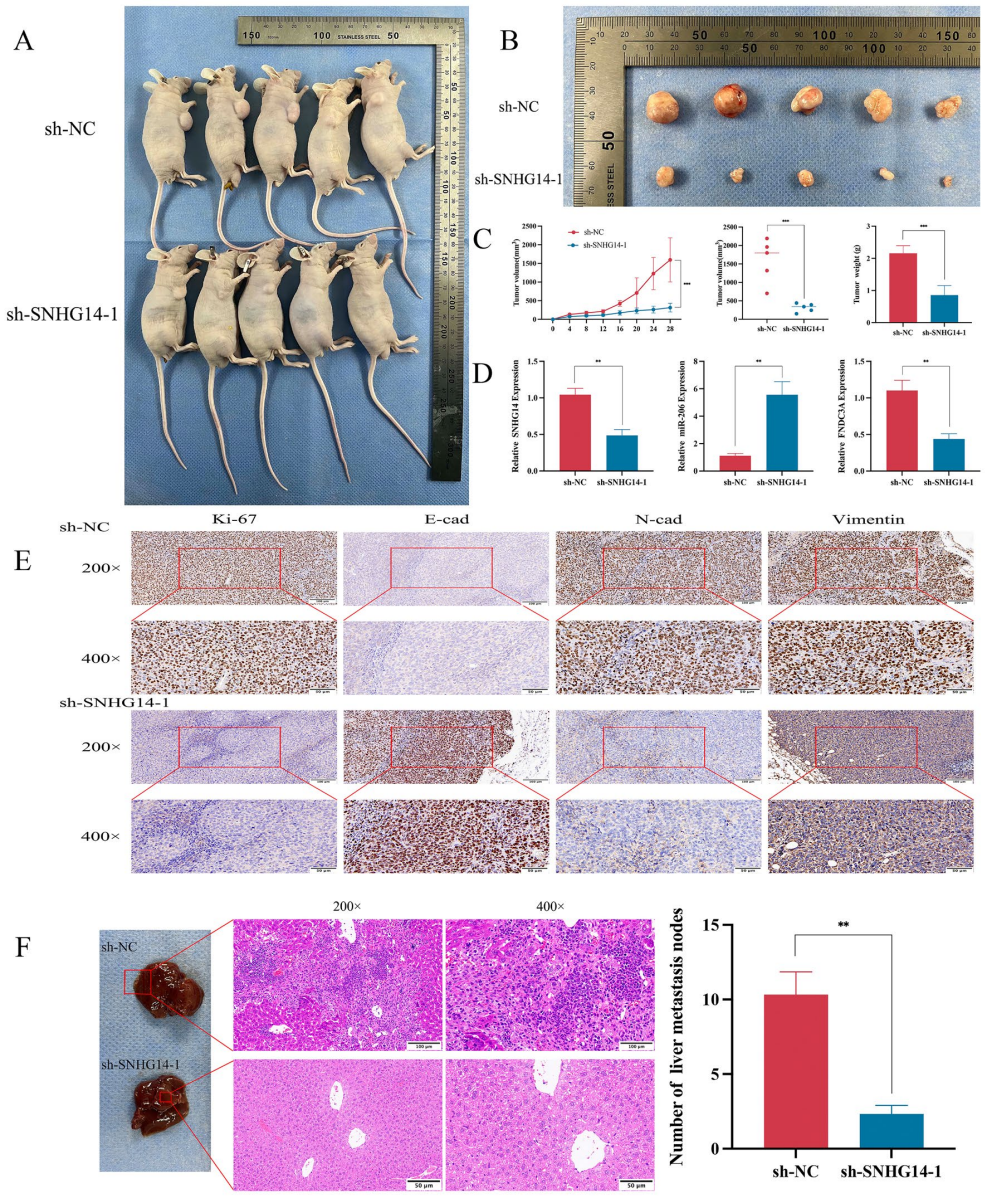
Depletion of SNHG14 inhibits tumour growth and metastasis in nude mouse cell xenografts. (A, B) Gross appearance and external whole‐body images of subcutaneous tumours from mice administered subcutaneous injection of sh‐NC and sh‐SNHG14‐1 GC cells. (C) Measure and count the tumour volumes and weights of the tumour. (D) The expression of SNHG14, miR‐206 and FNDC3A in the sh‐NC and sh‐SNHG14‐1 groups. (E) The protein levels of E‐cadherin, N‐cadherin, Vimentin, Ki‐67 and FNDC3A in tumours from indicated cells were estimated by IHC. (F) The HE staining of metastatic nodules in the livers of mice and the number of metastatic tumours in the sh‐NC and sh‐SNHG14‐1 groups *p < 0.05, **p < 0.01 and ***p < 0.001.

Tumour volume and weight were significantly reduced in the SNHG14 knockdown group compared to the control group (Figure 7A–C). After removing the tumour tissues, compared to the sh‐NC group, the tumours from the sh‐SNHG14‐1 group exhibited a decreased SNHG14 expression and elevated miR‐206 expression with a weakened FNDC3A expression. Our findings indicated that the downregulation of SNHG14 expression results in an upregulation of miR‐206 and a downregulation of FNDC3A. The findings were verified by our in vitro experimental mechanism (Figure 7D). Immunohistochemical staining was performed, indicating elevated levels of E‐cadherin within the SNHG14 knockdown group compared to the control group. The Ki‐67 levels, a protein indicating proliferative activity, as well as N‐cadherin, vimentin and FNDC3A, were significantly reduced (Figure 7E). These results follow those mentioned above in in vitro experiments. Furthermore, metastasis experiments were conducted in mice in vivo, suggesting that, compared to the control group, secondary tumours metastasising to the liver were diminished in the SNHG14 knockdown group (Figure 7F). Therefore, SNHG14 can facilitate the proliferation and metastasis of gastric cancer.

## 4 Discussion

Gastric cancer has a low early detection rate, and the cancer often advances to the middle or late stages before the symptoms become apparent [19]. Subsequent factors like metastasis and drug resistance lead to poorer survival rates among GC patients [20]. Genetic mutations are a significant cause impacting tumour progression, inducing abnormal and uncontrolled growth of tumour cells [21]. Mounting evidence indicates that long non‐coding RNAs (lncRNAs) are crucial in the genetic regulation regulating tumour proliferation, apoptosis and migration [22]. Wu's study suggests that SNHG26 is upregulated in GC and interacts with NCL, enhancing the proliferation and metastasis of GC by elevating c‐Myc translation [23]. Yu's studies have revealed that exosomal LOC85009 derived from lung adenocarcinoma (LUAD) cells can improve the sensitivity of DTX‐resistant LUAD cells to DTX while impeding the growth of xenograft tumours. LOC85009 binds to USP5 to impact USF1 and ATG5, thereby inhibiting the autophagy of LUAD cells [24]. The current research revealed that SNHG14 is overexpressed in GC cells, regulated by the transcription factor ZNF460. SNHG14 can directly mediate the proliferation and metastasis of GC using a novel ceRNA network associated with the SNHG14/miR‐206/FNDC3A axis.

SNHG14 is a significant promoter of tumour progression, with high expression levels seen nearly across all cancer types, highlighting its crucial role in oncogenesis [25]. Wang's study demonstrated that SNHG14 enables the proliferation and invasion of colorectal cancer cells by mediating the miR‐519b‐3p/DDX5 axis [26]. Zhang et al. showed that SNHG14 demonstrates low expression in colorectal cancer tissues, involving cancer progression and metastasis by competing with miR‐92b‐3p [27]. Furthermore, SNHG14 can upregulate PABPC1 via H3K27 acetylation, regulating the PTEN signalling pathway in hepatocellular carcinoma cells while promoting cell proliferation and angiogenesis [28]. Nevertheless, studies on SNHG14 in GC are relatively scarce. We have examined clinical samples and gastric cancer cells and observed that SNHG14 is highly expressed in GC, correlating with poor prognosis. The ROC curve analysis based on its expression indicates that SNHG14 has a good diagnostic effect for gastric cancer and can potentially become a diagnostic biomarker. Subsequent univariate and multivariate Cox regression analyses revealed that SNHG14 could be an independent prognostic factor for GC. Cellular functional experiments highlighted SNHG14 as an oncogenic factor promoting GC progression in cell lines and nude mice. In summary, SNHG14 could be a valuable biomarker and therapeutic target against gastric cancer.

Long non‐coding RNAs (lncRNAs) are intimately associated with their subcellular localization and functions [29]. When situated within the nucleus, lncRNAs can maintain the structural integrity of chromatin, controlling the transcription of genes while participating in the alternative splicing of mRNA [30]. In the cytoplasm, they are involved in signal transduction, post‐transcriptional regulation, translation and post‐translational modifications [31]. They assist in fulfilling the functions of these cellular components after localising to organelles [32]. The lncRNA subcellular localization mechanisms are closely associated with the RNA sequences and the proteins they interact with. The ceRNA hypothesis indicates that lncRNAs become molecular sponges for miRNAs, competing with mRNAs for miRNA binding, thereby suppressing miRNA regulation of target gene mRNAs [33]. This mechanism enables lncRNAs to participate in post‐transcriptional regulation of cellular processes, such as proliferation, invasion, migration, EMT and apoptosis. Sang's study on the biological functions of lncRNA SNHG14 in thyroid cancer cell development by targeting miR‐206 reveals that SNHG14 upregulation elevates miR‐206 levels, impacting the proliferation, invasion, apoptosis and EMT of thyroid cancer cells [34]. Nevertheless, the role of FNDC3A in gastric cancer and its relationship with microRNAs need to be investigated. Our research has depicted a negative correlation between FNDC3A expression and miR‐206 expression, with FNDC3A regulating GC progress.

Recently, the rapid advancement of second‐generation high‐throughput sequencing has facilitated the application of omics data, such as genomics, transcriptomics and epigenomics, to study lncRNAs in tumours [35]. Constructing ceRNA regulatory networks can provide new insights into the pathogenesis of GC by analysing the differential expression of miRNAs, lncRNAs and mRNAs between GC tissues or cells and normal gastric mucosa and corresponding cells [36]. Nevertheless, the principal methodologies employed in these studies depend on bioinformatics analysis techniques, necessitating further validation via basic experimental approaches. Our research identified a joint analysis of miRNA and mRNA expression in the SNHG14 negative control group. Moreover, the SNHG14‐silenced group facilitated the identification of the SNHG14/miR‐206/FNDC3A regulatory network. Furthermore, SNHG14 may play a critical role in the metastasis and EMT of GC via the TGF‐β/MAPK pathway. Tang's research indicates that lncRNA‐ATB enhances the invasion of TGF‐β‐induced glioma cells via the NF‐κB and P38/MAPK pathways [37]. Ge's study suggests that TRPC1/3/6 inhibition attenuates TGF‐β1‐induced EMT in GC through the Ras/Raf1/ERK signalling pathway [38]. Additional analyses with luciferase assays, RNA pull‐down assays and RIP assays have revealed that SNHG14 is a direct miR‐206 target, sponge‐adsorbing miR‐206 to mediate FNDC3A. Furthermore, the impact of this regulatory network on GC cell function has been corroborated using co‐transfection experiments. Thus, miRNAs can help reverse the pro‐oncogenic or tumour‐suppressive effects of lncRNAs or mRNAs. The results provide solid evidence for the regulatory role underlying the SNHG14/miR‐206/FNDC3A ceRNA network mechanism in the EMT of GC.

The study has endeavoured to explore the impact of transcription factors on SNHG14. Genes are transcribed and activated by regulating various factors [39]. The activation or silencing of specific promoters in the gene promoter region is crucial for gene expression [40]. Long non‐coding RNA LYPLAL1–DT is a tumour suppressor gene in triple‐negative breast cancer (TNBC). It has reduced expression during transcriptional regulation of the upstream transcription factor FOXO1, interacts with hnRNPK and suppresses the Wnt/β—catenin signalling pathway reactivation [41]. Zinc finger proteins (ZNFs) include zinc finger DNA‐binding domains that recognise and bind to the gene promoter region. ZNFs behave as transcription factors and regulate cellular functions, such as cell proliferation, differentiation, metabolism and immune responses [42]. Shao's research depicts that the transcription factor ZNF460 regulates COMMD7 and impacts acute myeloid leukaemia (AML) progression via the NF‐κB signalling pathway [43]. Currently, there are few reports on ZNF460 in GC. The combination of ZNF460 with the SNHG14 promoter has been verified using CHIP experiments. This demonstrates the regulation of SNHG14 by ZNF460 and provides new theoretical evidence to understand the transcription factor‐lncRNA axis.

## 5 Conclusion

**FIGURE 8:**
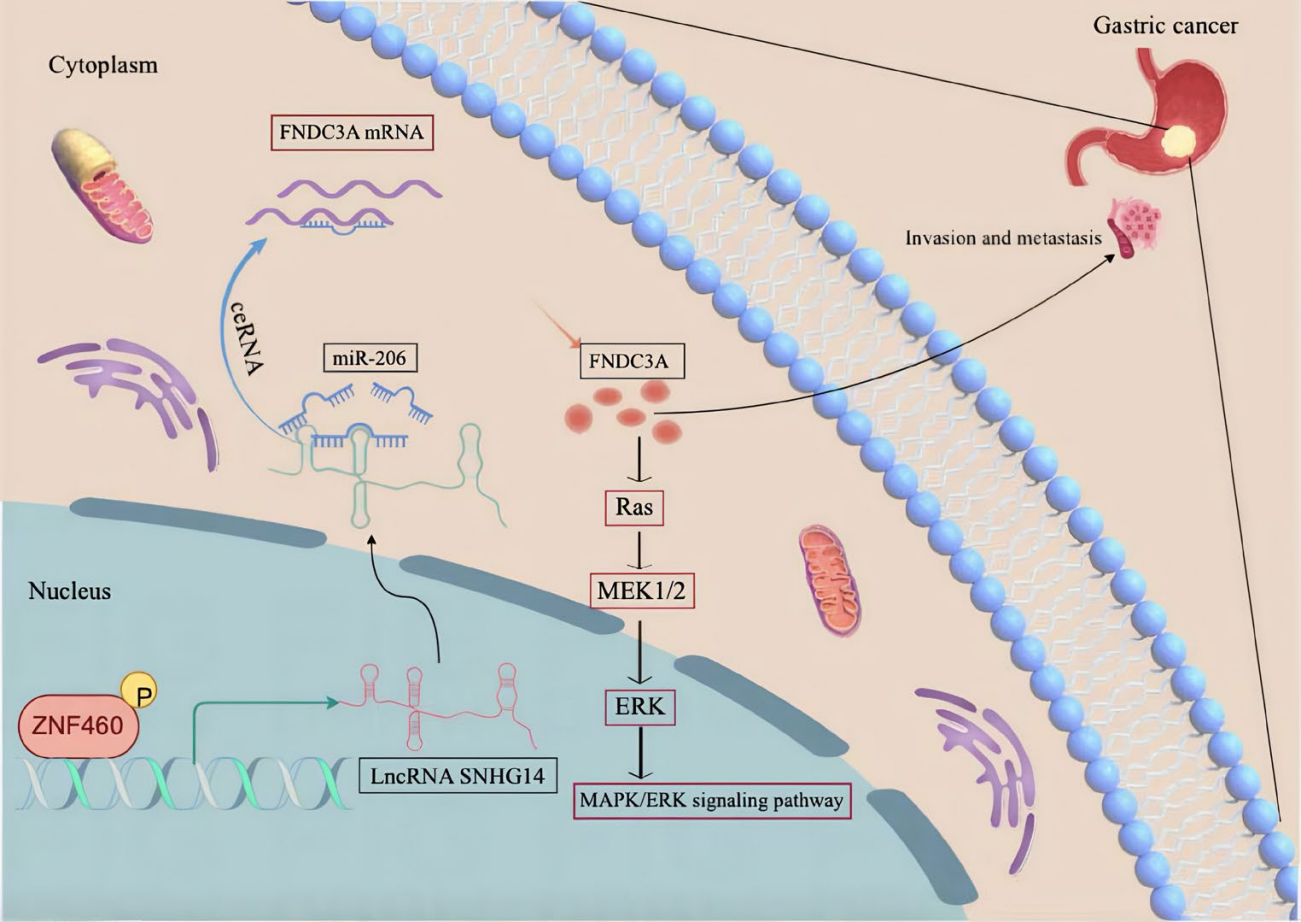
Scheme for the regulatory mechanism of lncRNA SNHG14 promotes GC metastasis.

Our findings suggest that SNHG14 is associated with a poor prognosis in GC and is mediated by the transcription factor ZNF460. Furthermore, the SNHG14/miR‐206/FNDC3A regulatory network influences the metastasis and EMT of GC, thereby providing new targets for diagnosis and therapy (Figure 8). Based on miRNA and mRNA sequencing data, we will investigate the novel mechanism through which SNHG14 targets mRNAs using RNA‐binding proteins.

## Author Contributions

**Bin Liu:** data curation (equal), formal analysis (equal), investigation (equal), methodology (equal), resources (equal), software (equal), validation (equal), visualization (equal), writing – original draft (equal). **Tingting Lu:** formal analysis (equal), investigation (equal), methodology (equal), resources (equal), software (equal), validation (equal), visualization (equal), writing – review and editing (equal). **Guangming Zhang:** formal analysis (equal), investigation (equal), methodology (equal), resources (equal), software (equal), validation (equal), writing – review and editing (equal). **Xiaohua Dong:** formal analysis (equal), resources (equal), software (equal), validation (equal), visualization (equal). **Miao Yu:** conceptualization (equal), data curation (equal), funding acquisition (equal), methodology (equal), project administration (equal), supervision (equal), validation (equal), writing – original draft (equal), writing – review and editing (equal). **Hui Cai:** conceptualization (equal), formal analysis (equal), funding acquisition (equal), methodology (equal), project administration (equal), supervision (equal), validation (equal), writing – original draft (equal), writing – review and editing (equal).

## Ethics Statement

All experiments performed with human samples were approved by the Gansu Provincial Hospital Ethics Committee (No. 2022–391). Written informed consent was obtained from all patients. All animal procedures were approved by the Laboratory Animal Science Center of Lanzhou University (No. MECI20240001).

## Conflicts of Interest

The authors declare no conflicts of interest.

## Supporting information


Figure S1.

Figure S2.

Table S1.

## Data Availability

The data and materials that support the findings of current study are available from the corresponding authors upon reasonable request.
