# Supplementary figures and images for: LncRNA SNHG14 Regulated by ZNF460 Promotes Gastric Cancer Progression and Metastasis by Targeting the miR‐206/FNDC3A Axis
Source: J Cell Mol Med. 2025 Jun 16;29(11):e70652. doi: 10.1111/jcmm.70652 (PMC12168236; doi:10.1111/jcmm.70652)

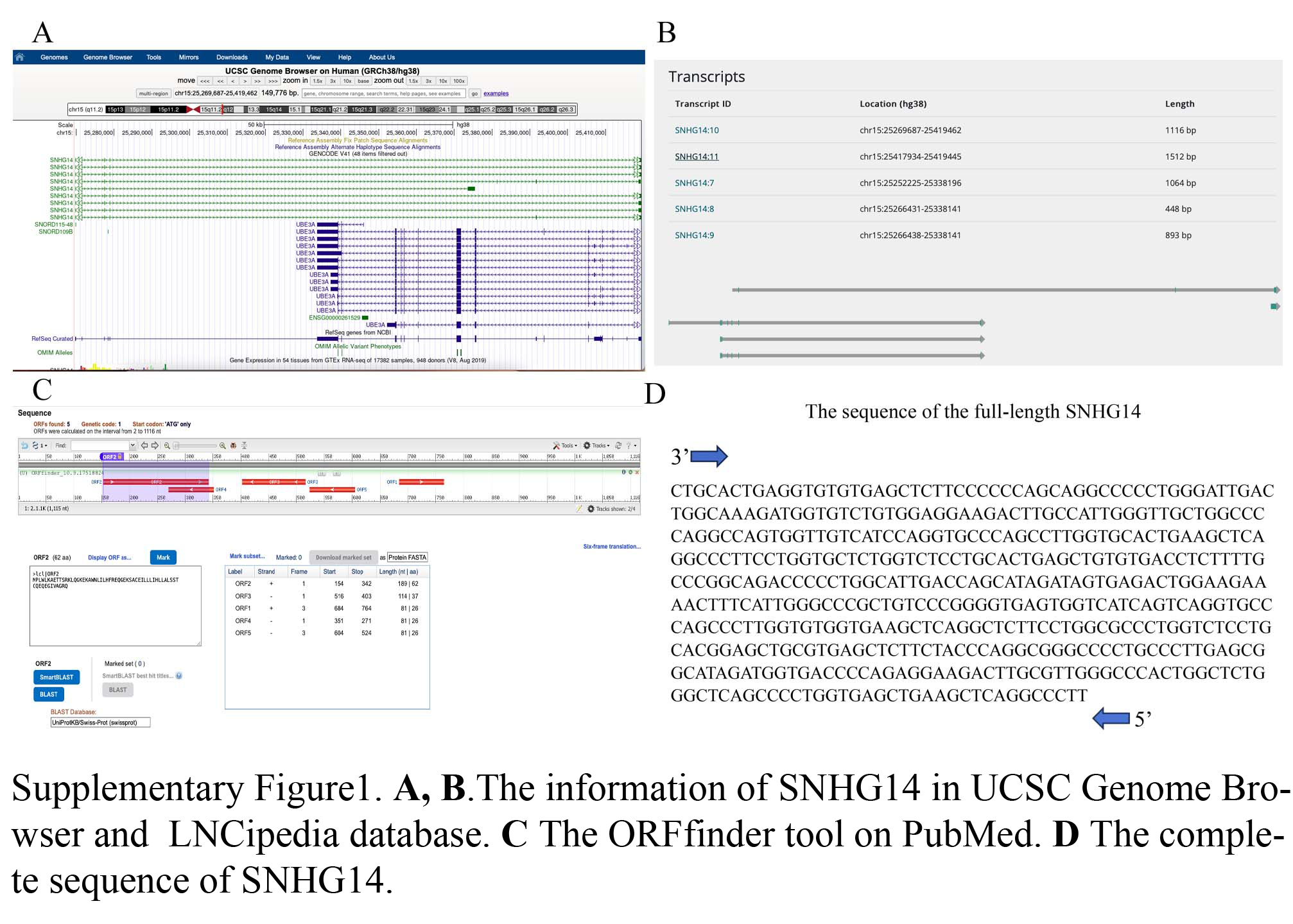

Supplement: Supplementary file 1 — Figure S1. [file JCMM-29-e70652-s003.tif]

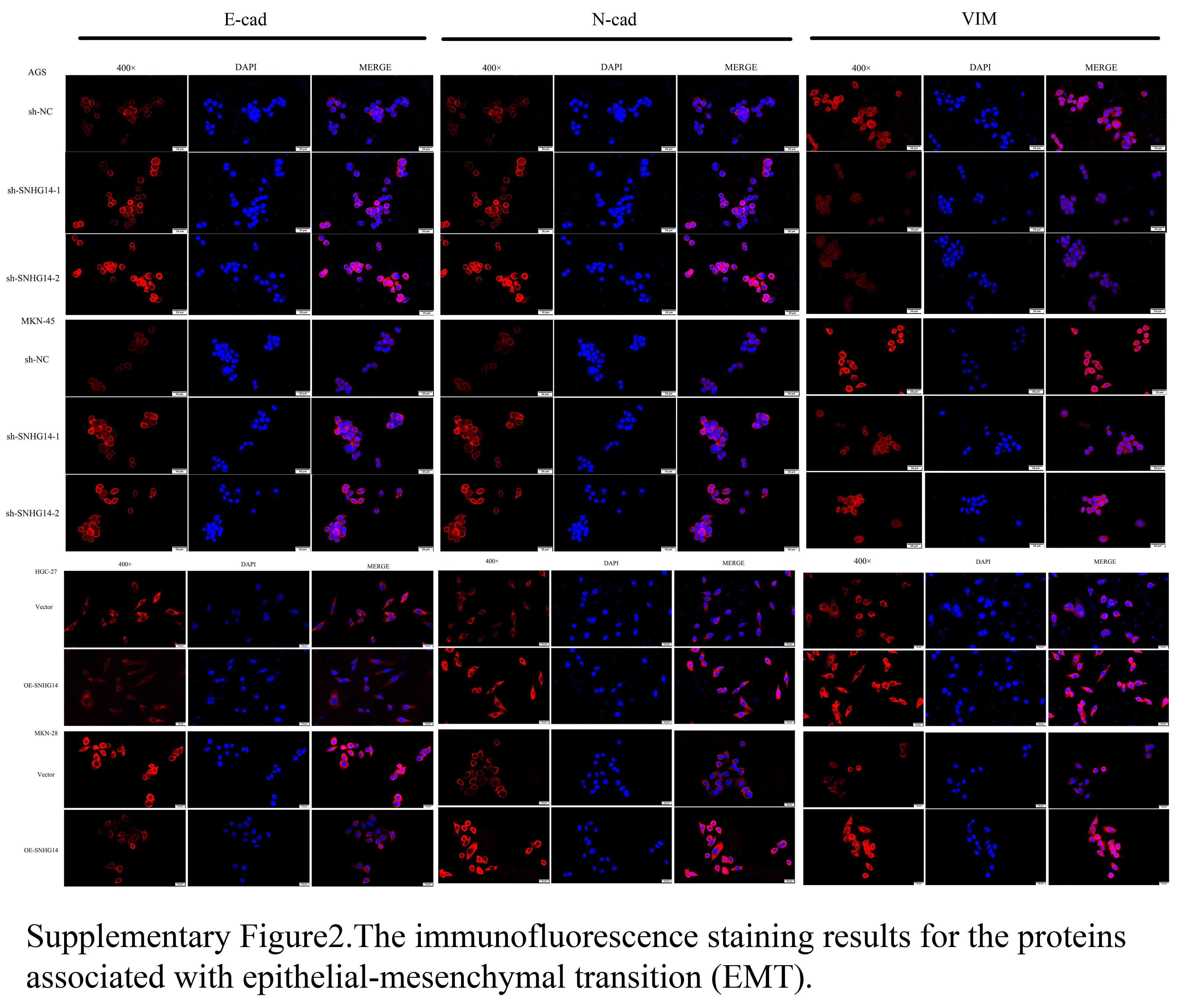

Supplement: Supplementary file 2 — Figure S2. [file JCMM-29-e70652-s002.jpg]
