# Supplementary material for: LncRNA SNHG14 Regulated by ZNF460 Promotes Gastric Cancer Progression and Metastasis by Targeting the miR‐206/FNDC3A Axis
Source: J Cell Mol Med. 2025 Jun 16;29(11):e70652. doi: 10.1111/jcmm.70652 (PMC12168236; doi:10.1111/jcmm.70652)
Supplement: Supplementary file 3 — Table S1. [file JCMM-29-e70652-s001.docx]

Sequences of Primer used in this study

| Primer name | primer sequence (5’-3’) |
| --- | --- |
| SNHG14- Forward | CCTTCCTGGTGCTCTGGTCTC |
| SNHG14- Reverse | TCTCACTATCTATGCTGGTCAATGC |
| miR-206- Forward | GCCCGCTGGAATGTAAGGAAGT |
| miR-206- Reverse | CCAGTGCAGGGTCCGAGGT |
| FNDC3A- Forward | CCCAAGAATATATTTTCACTACTCCAA |
| FNDC3A- Reverse | TTCACAAATGTGATCATTTACTTTCTC |
| NOTCH3- Forward | CGTCAGTGTGAACTCCTCTCC |
| NOTCH3- Reverse | AGATACCATGAGGGCCACAG |
| ZNF460- Forward | CTCATTCGACACTTCAACATCC |
| ZNF460- Reverse | GTGGATGCTAAAGTGTCGAATC |
| GADPH- Forward | ACCCAGAAGACTGTGGATGG |
| GADPH- Reverse | TTCTAGACGGCAGGTCAGGT |
| U6- Forward | TTATGGGTCCTAGCCTGAC |
| U6- Reverse | CACTATTGCGGGTCTGC |
| β-actin- Forward | TGGATATGCCCCACAGGTTA |
| β-actin- Reverse | GGAGGATGTGGAGAACGGTG |
| sh-NC sence | GTTCTCCGAACGTGTCACGT |
| sh-SNHG14-1 sence | CCCCGGTGGTGTTGGTATAAA |
| sh-SNHG14-2 sence | GACTCCCACCTAAGGGAATTA |
| sh-SNHG14-3 sence | AAGGGTGTGGTATACTAACAA |
| miR-NC | CAGUACUUUUGUGUAGUACAA |
| miR-206 inhibitor | CCACACACUUCCUUACAUUCCA |
| si-NC-sense | UUCUCCGAACGUGUCACGUTT |
| si-FNDC3A sence | GGAGCAACCAUCAAUAAAUTT |
| si-ZNF460 sence | GCGACAGCTGATGGTATTTGT |
| SNHG14-5’RACE | GAGACCAGAGCACCAGGAAGG |
| SNHG14-3’RACE | TTTTTTTTTTTTTTTTTTTTTTTTTVN |
| LncSNHG14 Promoter- Forward | GCTATTTGAGGAATGCGGCCTT |
| LncSNHG14 Promoter- Reverse | TATGTATTTCGAAAAATGATTAT |
